# Photo Quiz: Incidental cyst lesions during COVID-19 pneumonia admission

**DOI:** 10.1128/jcm.00494-24

**Published:** 2025-02-19

**Authors:** Alfredo Maldonado-Barrueco, Guillermo Ruiz-Carrascoso, Rosa de Miguel-Buckley, Susana Ayuela-García, Julio García-Rodríguez, Marta Mora-Rillo

**Affiliations:** 1 Clinical Microbiology and Parasitology Department, Hospital Universitario La Paz-Carlos III-Cantoblanco 221885 , Madrid, Spain; 2 IdiPAZ 638528 , Madrid, Spain; 3 Health Institute Carlos III, Madrid, Spain; 4 CIBERINFEC ISCIII, Instituto de Salud Carlos III 91837 , Madrid, Spain; 5 Imported Tropical Pathology, High Level Isolation Unit, Hospital Universitario La Paz, Madrid, Spain; 6 Infectious Diseases Unit, Internal Medicine Department, Hospital Universitario La Paz 16268 , Madrid, Spain; 7 General and Digestive Surgery, Hospital Universitario La Paz, Madrid, Spain; Mayo Clinic Minnesota, Rochester, Minnesota, USA

## PHOTO QUIZ 

**Fig 1:**
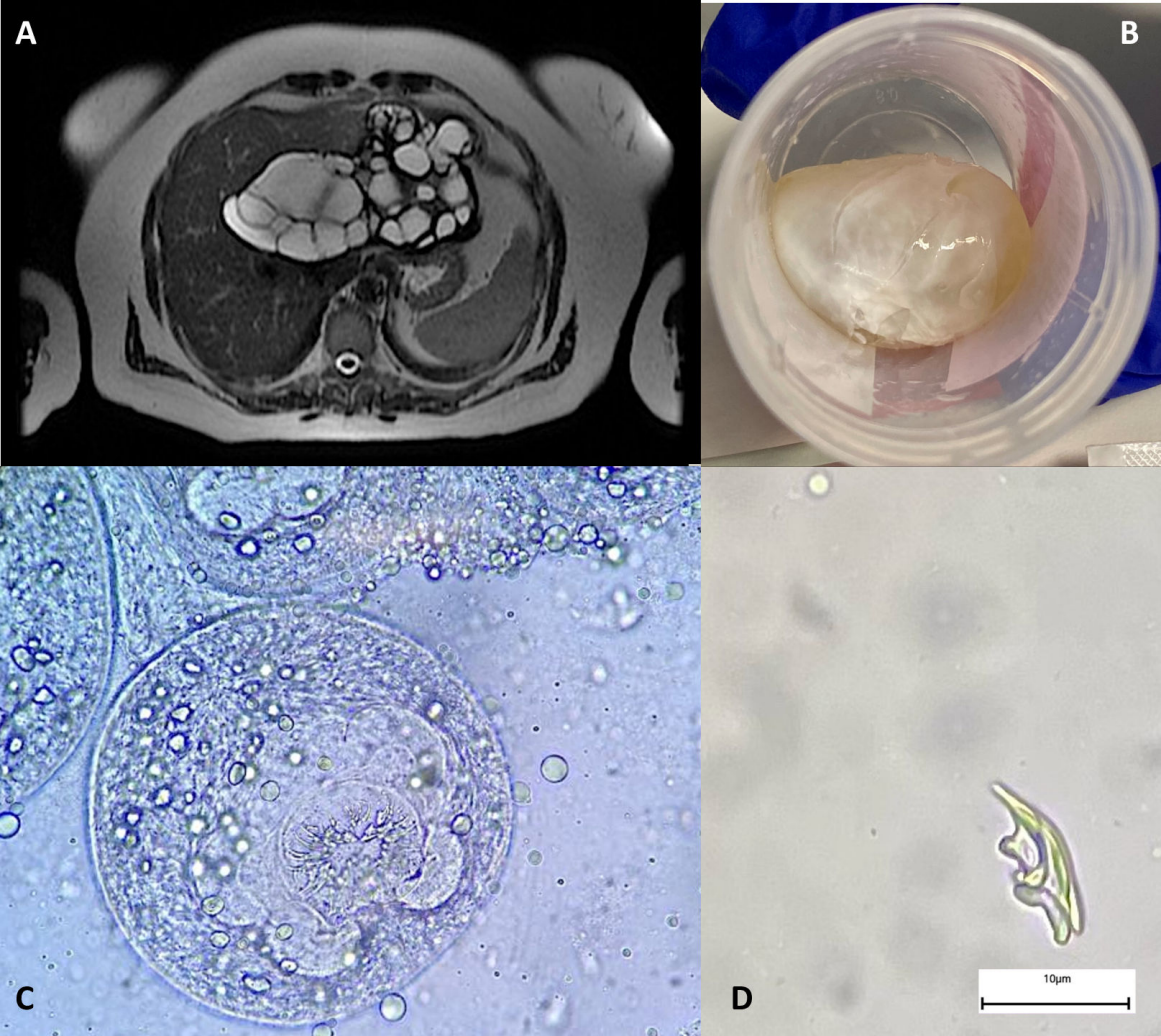
(A) Computed tomography scan of the liver shows confluent multitabulated space-occupying cystic lesions in “honeycomb” in the left hepatic lobe. (B) Intact hepatic cyst with wall obtained after surgery. (C) Direct wet mount microscopic findings of cyst aspirate (×400). (D) The object was observed in the wet mount of ×400.

A 46-year-old female with a known history of mild asthma was admitted to La Paz University Hospital in November 2020. The patient was positive for SARS-CoV-2 and had bilateral pneumonia. However, in the pulmonary computed tomography (CT) scan, incidental hepatic cystic lesions were identified in the upper abdomen. During admission, the patient received oxygen through a nasal cannula, molnupinavir (in the context of a COVID-19 clinical trial), and dexamethasone. The patient fully recovered and was discharged after 6 days of admission. Incidental hepatic cystic lesions were studied as outpatients 1 month later. She had lived in a rural area with contact with dogs during her childhood in Romania until moving to Madrid, Spain, in 2000. The blood count showed a total leukocyte count of 12.69 (reference range 3.90–10.20 × 10^3^/µL), differential leukocyte count with 73.5% neutrophils (reference range, 42%–77%), 12.1 lymphocytes (reference range 20%–44%) with increased eosinophils of 9.6% (reference range 0.5%–5.5%), and C-reactive protein of 192.4 (reference range <0.5 mg/L). The outpatient study included hepatic magnetic resonance imaging (MRI) and body CT scan. MRI and CT both showed confluent multivesicular space-occupying cystic lesions in the left hepatic lobe that penetrated the right hepatic lobe, compressing the middle suprahepatic vein (Fig. 1A). Cystic specimen was sent for microbiological and histological studies after surgery (Fig. 1B). Microscopic observation in the wet mount revealed free hooklets (Fig. 1C).

## ANSWER TO PHOTO QUIZ

Initially, the cyst lesions in the upper abdomen during the SARS-CoV-2 diagnosis were suggested to be biliary cystadenoma, cystadenoid carcinoma, or, less probably, cystic infectious lesions. Later, during the outpatient study, the cystic lesions in “honeycomb” were in stage II of cystic echinococcosis (CE2), consisting of active multiple daughter vesicles and septa with mixed echogenicity, as per the classification proposed by the World Health Organization Informal Working Group according to the activity of the cystic lesion and based on ultrasonographic findings (1). The hydatid cyst occupied the entire left liver and penetrated the right liver lobe. Serology showed a positive high titer of indirect hemagglutination (1:5,120), positive anti-*Echinococcus* IgG by enzyme-linked immunosorbent assay 1.85 (reference value >1.1) (Novalisa® Echinococcus IgG, NovaTech, Germany), and positive specific anti-*Echinococcus* IgG4 3.00 (reference value >1.1). The subisotype responses against CE1, CE2, and CE3 cyst stages are mainly IgG4, whereas IgG1, IgG2, and IgG3 responses predominate against CE4 and CE5 cysts. Moreover, IgG2 and IgG4 levels could be related to cyst stages, disease evolution, and echinococcosis relapses (2). The diagnosis cannot be serological alone because it depends on the type of assay, the immune status of the patient, and the location and stage of the hydatid cysts (3).

Despite the broad radiologic differential diagnosis, the positive serology together with compatible epidemiological past history (patient from Romania) made liver echinococcosis the most probable diagnosis; therefore, she was initiated with preoperative albendazole prophylaxis until hepatic surgery. Left open hepatectomy was performed without any aperture or spillage of the cyst into the abdominal cavity. Fistulization or rupture of the cyst, usually into the peritoneal or pleural cavities, can lead to both the spread of infection to other organs and the development of anaphylactic reactions (3). A minor postoperative complication was a biliary leak that resolved without further surgery. Albendazole 400 mg twice daily was maintained for 3 months after surgery.

Cystic specimens were sent for microbiological and histological studies (Fig. 1B). Microscopic observation of one hydatid vesicle showed protoscolices with armed rostellum (Fig. 1C) and a free hooklet suggestive of *Echinococcus* spp. (Fig. 1D).

Humans are aberrant intermediate hosts in echinococcosis and become infected by ingesting eggs through contaminated food or water, by fomites, or by direct contact with dogs. Oncospheres are released in the intestine, migrate through the circulatory system, and hydatid cysts develop in different organs, mainly in the liver and lungs (4). However, the presence of eggs in feces is not present in humans with echinococcosis.

Infected humans may remain asymptomatic for years, and the diagnosis may be incidental, such as in our case. The WHO also included Romania in the list of echinococcosis endemic countries (5). In Romania, the prevalence of echinococcosis was 7.2 per 100,000 (6). However, there is probably substantial underreporting of the disease because of the higher epidemiological risk factors of the population. In Romania, approximately 45.7% of the population lives in rural areas, and 30% works in the agricultural sector (7).

The patient is followed up every 6 months with abdominal ultrasound and serology to detect early signs of recurrence (8). Three years after diagnosis and treatment, the patient is asymptomatic, with decreasing IgG4 levels and stable hemagglutination titers, without evidence of recurrence on abdominal ultrasound.

## References

[1] WHO Informal Working Group. 2003. International classification of ultrasound images in cystic Echinococcosis for application in clinical and field epidemiological settings. Acta Trop 85:253–261. doi:10.1016/S0001-706X(02)00223-1 12606104

[2] Manzano-Román R, Sánchez-Ovejero C, Hernández-González A, Casulli A, Siles-Lucas M. 2015. Serological diagnosis and follow-up of human cystic Echinococcus infection: a new hope for the future? Biomed Res Int 2015:428205. doi:10.1155/2015/428205 26504805 PMC4609352

[3] Bhutani N, Kajal P. 2018. Hepatic Echinococcosis: a review. Ann Med Surg (Lond) 36:99–105. doi:10.1016/j.amsu.2018.10.032 30450204 PMC6226561

[4] McManus DP, Zhang W, Li J, Bartley PB. 2003. Echinococcosis. Lancet 362:1295–1304. doi:10.1016/S0140-6736(03)14573-4 14575976

[5] WHO. 2024. Echinococcosis. Available from: https://www.who.int/news-room/fact-sheets/detail/echinococcosis. Retrieved 23 Mar 2024.

[6] Tamarozzi F, Akhan O, Cretu CM, Vutova K, Akinci D, Chipeva R, Ciftci T, Constantin CM, Fabiani M, Golemanov B, Janta D, Mihailescu P, Muhtarov M, Orsten S, Petrutescu M, Pezzotti P, Popa AC, Popa LG, Popa MI, Velev V, Siles-Lucas M, Brunetti E, Casulli A. 2018. Prevalence of abdominal cystic Echinococcosis in rural Bulgaria, Romania, and Turkey: a cross-sectional, ultrasound-based, population study from the HERACLES project. Lancet Infect Dis 18:769–778. doi:10.1016/S1473-3099(18)30221-4 29793823

[7] Paduraru AA, Lupu MA, Sima L, Cozma GV, Olariu SD, Chiriac SD, Totolici BD, Pirvu CA, Lazar F, Nesiu A, Mihu AG, Cumpanas AA, Cretu OM, Olariu TR. 2023. Echinococcosis in hospitalized adult patients from Western Romania: 2007-2022. Microorganisms 11:2388. doi:10.3390/microorganisms11102388 37894047 PMC10609572

[8] Bocanegra C, Álvarez-Martínez MJ, Arsuaga Vicente M, Belhassen-García M, Chamorro Tojeiro S, Camprubí-Ferrer D, Fernández Soto P, García Vázquez E, Herrador Ortiz Z, Martín O, Muro A, Pérez Arellano JL, Reguera Gómez M, Salas-Coronas J, Salvador F, Sotillo Gallego J, Sulleiro E, Torrús Tendero D, Velasco Arribas M, Rodríguez Guardado A. 2023. Executive summary consensus statement of imported diseases group (GEPI) of the Spanish society of infectious diseases and clinical microbiology (SEIMC) and the Spanish society of tropical medicine and international health (SETMSI), on the diagnostic and treatment of imported schistosomiasis. Enferm Infecc Microbiol Clín 41:505–512. doi:10.1016/j.eimc.2023.02.004 37230838

